# Novel nutritional indicator as predictors among subtypes of lung cancer in diagnosis

**DOI:** 10.3389/fnut.2023.1042047

**Published:** 2023-01-26

**Authors:** Haiyang Li, Zhangkai J. Cheng, Zhiman Liang, Mingtao Liu, Li Liu, Zhenfeng Song, Chuanbo Xie, Junling Liu, Baoqing Sun

**Affiliations:** ^1^Department of Clinical Laboratory, National Clinical Research Center of Respiratory Disease, Guangzhou Institute of Respiratory Health, First Affiliated Hospital of Guangzhou Medical University, Guangzhou Medical University, Guangzhou, China; ^2^Department of Allergy and Clinical Immunology, National Clinical Research Center of Respiratory Disease, Guangzhou Institute of Respiratory Health, First Affiliated Hospital of Guangzhou Medical University, Guangzhou Medical University, Guangzhou, China; ^3^Cancer Center, Sun Yat-sen University, Guangzhou, China

**Keywords:** lung cancer subtypes, tumor nutrition, machine learning, nutritional indicators, cancer prediction

## Abstract

**Introduction:**

Lung cancer is a serious global health concern, and its subtypes are closely linked to lifestyle and dietary habits. Recent research has suggested that malnutrition, over-nutrition, electrolytes, and granulocytes have an effect on the development of cancer. This study investigated the impact of combining patient nutritional indicators, electrolytes, and granulocytes as comprehensive predictors for lung cancer treatment outcomes, and applied a machine learning algorithm to predict lung cancer.

**Methods:**

6,336 blood samples were collected from lung cancer patients classified as lung squamous cell carcinoma (LUSC), lung adenocarcinoma (LUAD), and small cell lung cancer (SCLC). 2,191 healthy individuals were used as controls to compare the differences in nutritional indicators, electrolytes and granulocytes among different subtypes of lung cancer, respectively.

**Results:**

Our results demonstrated significant differences between men and women in healthy people and NSCLC, but no significant difference between men and women in SCLC patients. The relationship between indicators is basically that the range of indicators for cancer patients is wider, including healthy population indicators. In the process of predicting lung cancer through nutritional indicators by machine learning, the AUC of the random forest model was as high as 93.5%, with a sensitivity of 75.9% and specificity of 96.5%.

**Discussion:**

This study supports the feasibility and accuracy of nutritional indicators in predicting lung cancer through the random forest model. The successful implementation of this novel prediction method could guide clinicians in providing both effective diagnostics and treatment of lung cancers.

## 1. Introduction

Nutritional changes, such as malnutrition and drastic changes in biomarkers, are commonly observed in otherwise healthy populations with cancer patients (1, 2). Metabolic and nutritional alterations can have a profound impact on survival and recovery in cancer patients, potentially leading to other complications (3). Calle et al. (4) have examined the role of both overnutrition and malnutrition in cancer development, and their interactions with nutrition indicators. Traditional markers, such as albumin (ABL) (5), total protein (TP) (6), total cholesterol (TCH) (7), glucose (GLU) (8), lactate dehydrogenase (LDH) (9), electrolytes (10), and granulocytes (11), are often used to evaluate the nutritional status of cancer patients (12–16), with ALB and TP being the most commonly used indicators for assessing nutritional status (17, 18). These have been extensively studied by Lv et al. (19) and Ikeda et al. (20) to both predict cancer occurrence and monitor the prognosis of cancer patients. Furthermore, Shibata (21), Bayne et al. (22), and Popescu and Stanescu (23) have demonstrated that electrolytes, granulocytes, and trace elements have a critical role in tumor development. Additionally, Zitvogel et al. (24) highlighted the effect of the leukocyte family on both quantitative and qualitative aspects of nutrition, and its influence on pro-inflammatory carcinogenic or anti-cancer immune responses. Despite this, there are few studies combining these markers to predict and analyze the development of lung cancer. Thus, combining traditional nutritional markers, electrolytes, and granulocytes into a novel nutrition index set can be used to develop a statistical model to more accurately depict the development and prediction of cancer.

Lung cancer is the leading cause of cancer-related mortality worldwide, accounting for 18.0% of all cancer deaths (25). It is largely attributed to poor lifestyle habits, dietary structure, genetic predisposition, air pollution, smoking, and excessive alcohol consumption (26–30). The two major subtypes of lung cancer are small cell lung cancer (SCLC) and non-small cell lung cancer (NSCLC). NSCLC represents the majority of lung cancer cases, and is composed mainly of lung squamous cell carcinoma (LUSC) and lung adenocarcinoma (LUAD) (25, 31). The 5-year survival rate for NSCLC is about 60% (32, 33); however, the 5-year survival rate for SCLC is lower, at 5–10% (34, 35). In recent years, research has focused on the use of nutritional indicators to predict lung cancer (36–38). Thus, we postulate that, with the integration of previous studies, the application of combined nutritional indicators might yield better prediction results.

**Figure 1:**
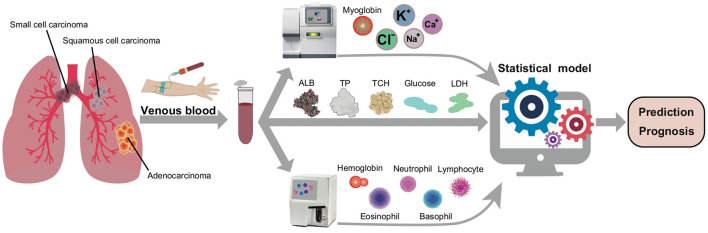
The scheme of cancer nutrition analyzing. The blood samples were performed through 6,336 different subtypes of lung cancer patients and about 2,191 healthy persons. After that, determine the electrolytes, nutritional indicators and granulocyte ratios and import the parameters into the statistical model to systematically predict the diagnosis and prognosis of cancer.

Here, we collected morning fasting venous blood samples from 6,336 people belonging to different subtypes of lung cancer, and 2,191 healthy persons formed the control group. Figure 1 presents the routine biochemical and specific indicators measured in both the experimental and the control groups. Subsequently, the Random Forest (RF) machine learning algorithm was applied to the collected blood sample index data in order to construct a Receiver Operating Characteristic (ROC) spectrum and calculate the area under the curve (AUC) to distinguish between normal and cancerous conditions. Furthermore, correlation analysis was conducted between the subtype indicators to identify the differences in lung cancer subtypes and the performance of nutrition-related indicators across different lung cancer subtypes. Finally, nutritional indicators, electrolytes, and white blood cell family data were combined to predict and diagnose lung cancer subtypes with higher accuracy and speed, using statistical models and machine learning algorithms.

## 2. Materials and methods

### 2.1. Patient population

This study included 6,339 patients who underwent curative surgery for subtypes of lung cancer from July 2017 to July 2022 in the affiliated hospital of Guangzhou Medical University. Venous blood samples were collected from these patients, who were medically and radiologically confirmed to have lung cancer subtypes, using heparin as an anticoagulant. The specimen was stored at 4°C, with 3,000 rpm centrifuged within 30 mins of collection, and the supernatant cleansed for packing before being stored at −80°C. Furthermore, a random selection of 2,191 blood samples were collected from healthy individuals aged 50–70 as controls shown in Table 1, after they had provided informed consent to participate in this study.

**Table 1:** The distribution of the number of subtype medical records of lung cancer.

	**Health**	**LUAD**	**LUSC**	**SCLC**
N	2,191	3,903	1,490	943
Male	1,139 (52%)	2,534 (65%)	1,144 (77%)	679 (72%)
Female	1,052 (48%)	1,369 (35%)	346 (23%)	264 (28%)
Age	60.3 ± 6.6	62.7 ± 8.0	63.1 ± 8.6	61.3 ± 7.2

### 2.2. Patients data acquisition

The patient's blood biochemistry data was collected via the LABOSPECT 006 Automatic Biochemical Analyser (Hitachi, Ltd., Tokyo, Japan) for the detection of ionic lipids, proteins, enzymes, hormones, and other metabolites. Additionally, the patient's granulocytes were counted using the Coulter AcT 5diff AL (Autoloader) Hematology Analyser (Beckman Coulter, Ltd, USA).

### 2.3. Statistical analyses

For the processing of overall data, we employed machine learning techniques such as k-Nearest Neighbors (KNN), t-Distributed Stochastic Neighbor Embedding (t-SNE) and Receiver Operating Characteristic (ROC). Furthermore, the differential analyses between biochemical indicators were conducted with the limma tool package (version 3.52; https://bioconductor.org/packages/limma), developed by Ritchie et al. (39). Continuous variables are presented as mean ± standard deviation (SD) and categorical variables are represented through numbers (percentage) or visualizations using R Studio and Python Programming.

The K-Nearest Neighbor (KNN) algorithm is a widely used classification technique that relies on measuring the distance between different feature values (40). Specifically, the Euclidean distance, which is calculated according to the following formula:


(1) $$\begin{array}{c} d\left(x,y\right)=\sqrt{\overset{n}{\underset{i=1}{\sum }}{\left(x_{i}-y_{i}\right)}^{2}} \end{array}$$

The classification results are sorted according to the increasing relationship of Euclidean distance and can be obtained using the Kknn" package (version 1.3.1) in R Programming, which is available at https://cran.r-project.org/web/packages/kknn/.

Secondly, the data after preliminary classification were visualized using the tsne package (version 0.1-3.1; https://cran.r-project.org/web/packages/tsne) in R Studio. The t-SNE algorithm is designed to convert the distance to a conditional probability in order to capture the similarity between points (41). Then, it uses the Kullback-Leibler divergence to measure the similarity between the high-dimensional points and the corresponding low-dimensional points. Finally, it minimizes the difference between the original high-dimensional points and the low-dimensional points by iterative steps (42).

We employed the Receiver Operating Characteristic (ROC) curve to evaluate and compare the efficacy of diagnostic models and to ascertain whether they are of practical value (43, 44). Moreover, we utilized the “pROC” package (version 1.18.0; https://cran.r-project.org/web/packages/pROC/) to visualize the ROC curve and the Area Under the Curve (AUC), with the latter serving as the key index. AUC is used to assess whether positives are ranked higher than negatives and is generally computed using the following formula:


(2) $$\begin{array}{c} AUC\left(f\right)=\frac{\sum_{t_{0}\in D^{0}}\sum_{t_{1}\in D^{1}}1\left[f\left(t_{0}\right)<f\left(t_{1}\right)\right]}{\left|D^{0}|\cdot |D^{1}\right|} \end{array}$$

where, **1**[*f*(*t*_0_) < *f*(*t*_1_)] denotes an indicator function which returns 1, if *f*(*t*_0_) < *f*(*t*_1_) otherwise returns 0; $D^{0}$ is the set of negative examples, and $D^{1}$ is the set of positive examples. After performing the ROC curve analysis of all biochemical and nutrition-related indexes, the ones with the highest AUC scores were selected for further analysis.

The correlation coefficient can be calculated using the following formula:


(3) $$\begin{array}{c} s_{xy}=\frac{\sum_{i=1}^{n}\left(X_{i}-\bar{X}\right)\left(Y_{i}-Ȳ\right)}{n-1} \end{array}$$

where *S*_*xy*_ represents the co-variance between the samples, and *S*_*x*_ and *S*_*y*_ represent the sample standard deviations of *x* and *y* respectively. The denominator of the formula is scaled by *n* − 1 due to it being a sample variance and a sample standard deviation. Additionally, the calculation formula for *S*_*x*_ sample standard deviation is:


(4) $$\begin{array}{c} s_{x}=\sqrt{\frac{\sum {\left(x_{i}-\bar{x}\right)}^{2}}{n-1}} \end{array}$$

The correlation coefficient can range from −1 to 1, where −1 is a perfect negative correlation and 1 is a perfect positive correlation. A correlation coefficient closer to 0 indicates a weaker correlation. All the indices were analyzed and calculated for this purpose.

### 2.4. Modeling of predictive models

Our model utilizes Bayes' theorem for classification and assumes that the classification is independent of the predictors. Naive Bayes is an ideal model for large datasets, and is capable of performing well in complex scenarios. To further analyze the data, we normalized 15 nutritional indicators and divided them into a training set (70%) and a verification set (30%) *via* random sampling. As Octaviani and Rustam (45) noted, the number of RF model training sets has a positive correlation with the prediction accuracy. To achieve optimal results, we developed an RF model using Python (version 3.7; http://www.Python.org) with the sklearn library (version 1.1.2; https://scikit-learn.org/stable/). The GridSearchCV9D module was used to adjust the parameters of the RF model, and approximately 200 trees with 15 variables were randomly selected for each tree, with a maximum depth of 50. We collected the results, selected the most performant model, and measured the prediction accuracy on the test set. Additionally, the model was optimized for the number of variables selected for each tree. To prevent the RF model from overfitting and to maintain the stability and practicality of the model, cross-validation was used during the parameter adjustment process. Discrimination performance was assessed based on the ROC curve and the corresponding AUC value.

### 2.5. Data visualization

Data acquisition and statistical analyses were performed using R Core Team version 4.2.1 and Python 3.7. The optimization of color and typesetting was completed through Adobe Illustrator (https://www.adobe.com). Column charts and box diagrams were drawn by the “matlibplot” Python package (version 3.5; https://matplotlib.org/). The visualization of pair plots was done using the “seaborn” Python package (version 0.11.2; https://seaborn.pydata.org/). The volcano plot was visualized using the “ggplot2” package (version 3.3.6; https://cran.r-project.org/web/packages/ggplot2) in R. A heat scatter was created using the “LSD” package (version 4.1-0; https://cran.r-project.org/web/packages/LSD) in R. Furthermore, the Circos plot was assisted by the “circlize” package (version 0.4.15; https://cran.r-project.org/web/packages/circlize) and TBTools software (version 1.0987657; https://github.com/CJ-Chen/TBtools/releases). The differences between male and female medical records of different cancers were compared visually using the “beanplot” package (version 1.3.1; https://cran.r-project.org/web/packages/beanplot/). Finally, the correlation coefficients between the data indices in the study were visualized by the “ggcorrplot” package (version 0.1.3; https://cran.r-project.org/web/packages/ggcorrplot/).

## 3. Results

### 3.1. The demographic characteristics of all patients

In this study, the majority of patients with lung cancer were aged 62.4 ± 7.9, so we mainly randomly selected individuals between the ages of 50 and 70 from the healthy population for comparison. And the Table 1 shows the distribution of different types of lung cancer. The table reveals that the age of the healthy population is 60.3 ± 6.6 and each subtype of cancer patients are mostly within this interval, i.e., LUAD: 62.7 ± 8.0, LUSC: 63.1 ± 8.6, SCLC: 61.3 ± 7.2. This study also shows that males are significantly more affected by all types of lung cancer than females. Furthermore, the results are consistent with the incidence rate of lung cancer subspecies, as the number of SCLC patients is much smaller than that of NSCLC patients.

**Figure 2:**
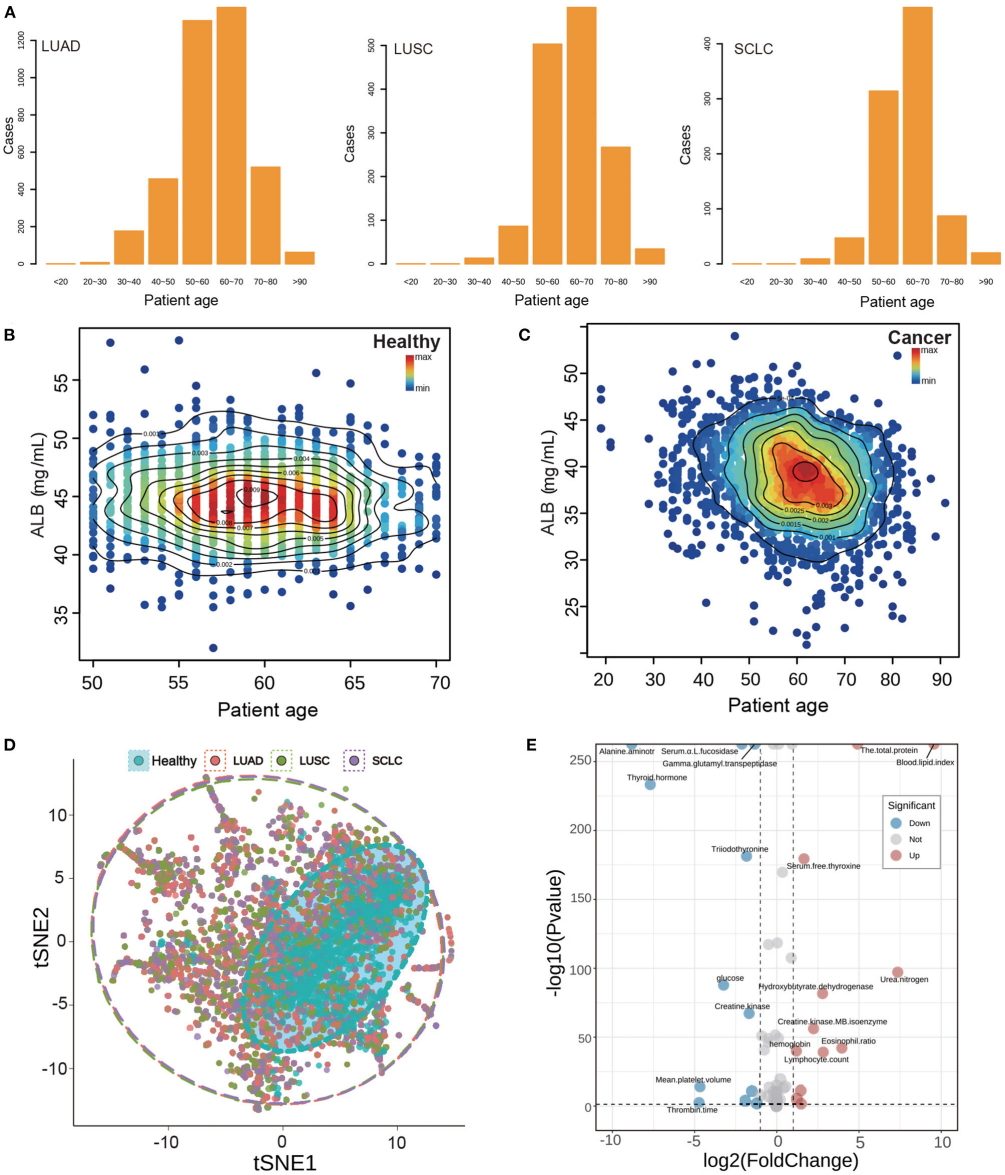
Basic information and index analysis of lung cancer patients. **(A)** The age distribution of subtype of lung cancer patients. **(B, C)** The ALB density scatters plot of healthy population and lung cancer patients. **(D)** The tSNE plot of the whole biochemical index of lung cancer and healthy population. Points of different colors represent different types of patients, and dotted coils represent different types of concentrated areas. **(E)** The volcano plot of the whole biochemical index of lung cancer and healthy population. The distribution of up-regulation and down-regulation trends of the index points with *P* < 0.05 and fold change more than 1 were shown, respectively.

The age distribution of patients with subtype lung cancer, as shown in Figure 2A, resembles that of the healthy population, with a peak concentration of individuals between 50 and 70 years of age. To further visualize the difference between these two groups, a scatter diagram of biochemical indicators was plotted. This revealed that the age range of the healthy population was confined to 50–70 years old (Figure 2B), whereas the biochemical indicators of lung cancer patients were found to span all age ranges (Figure 2C), with the majority of individuals concentrated in the same age bracket as the healthy population (50–70 years old).

### 3.2. Analysis of biochemical indexes of lung cancer

Nutritional indicators are closely related to the metabolism of individuals. Albumin (ALB) is often used as a marker to assess tumor development and prognosis (13). We studied the distribution of albumin and patient age (Figures 2B–D). The ALB content in healthy individuals was mainly concentrated at 45 mg/ml, while that in cancer patients was relatively low at 45 mg/ml. There was a significant difference between healthy and cancer patients. These results suggest that albumin may be a useful indicator for assessing tumor development and prognosis.

In this study, we analyzed more than 50 indicators of blood samples and found it difficult to accurately classify the analysis of multiple indicators by ordinary analysis methods. Therefore, we carried out t-SNE dimension reduction analysis for all indicators in order to show their expression in low dimensions. The results, shown in Figure 2B, indicate that different colors represent the distribution of two-dimensional indicators of different subtypes of lung cancer patients. The red color indicates the distribution of indicators of LUAD patients, the green color indicates the distribution of indicators of LUSC patients, and the purple color indicates the distribution of indicators of SCLC patients. Additionally, the dotted circle around the data points represents the range of data distribution. From the results, we observed that the healthy population is generally distributed inside cancer, which demonstrates that the normal indicators of the healthy population are covered by the indicators of cancer patients, indicating that the indicators of cancer patients are more disorderly and have a wide coverage. Furthermore, we can also observe from the distribution of 15 specific research indicators in Supplementary Figure S4 that the indicators of the healthy population are generally covered by the cancer population, which is consistent with the results of t-SNE. Additionally, we conducted a difference analysis of all biochemical indicators and showed the difference between the healthy population and the lung cancer population via a volcano map. The results are displayed in Figure 2E, which shows a fold change factor of 2 times and a significant *P* interval of 0.05; with 12 indicators up-regulated and 12 indicators down-regulated.

### 3.3. Correlation analysis and difference analysis of nutritional indexes

**Figure 3:**
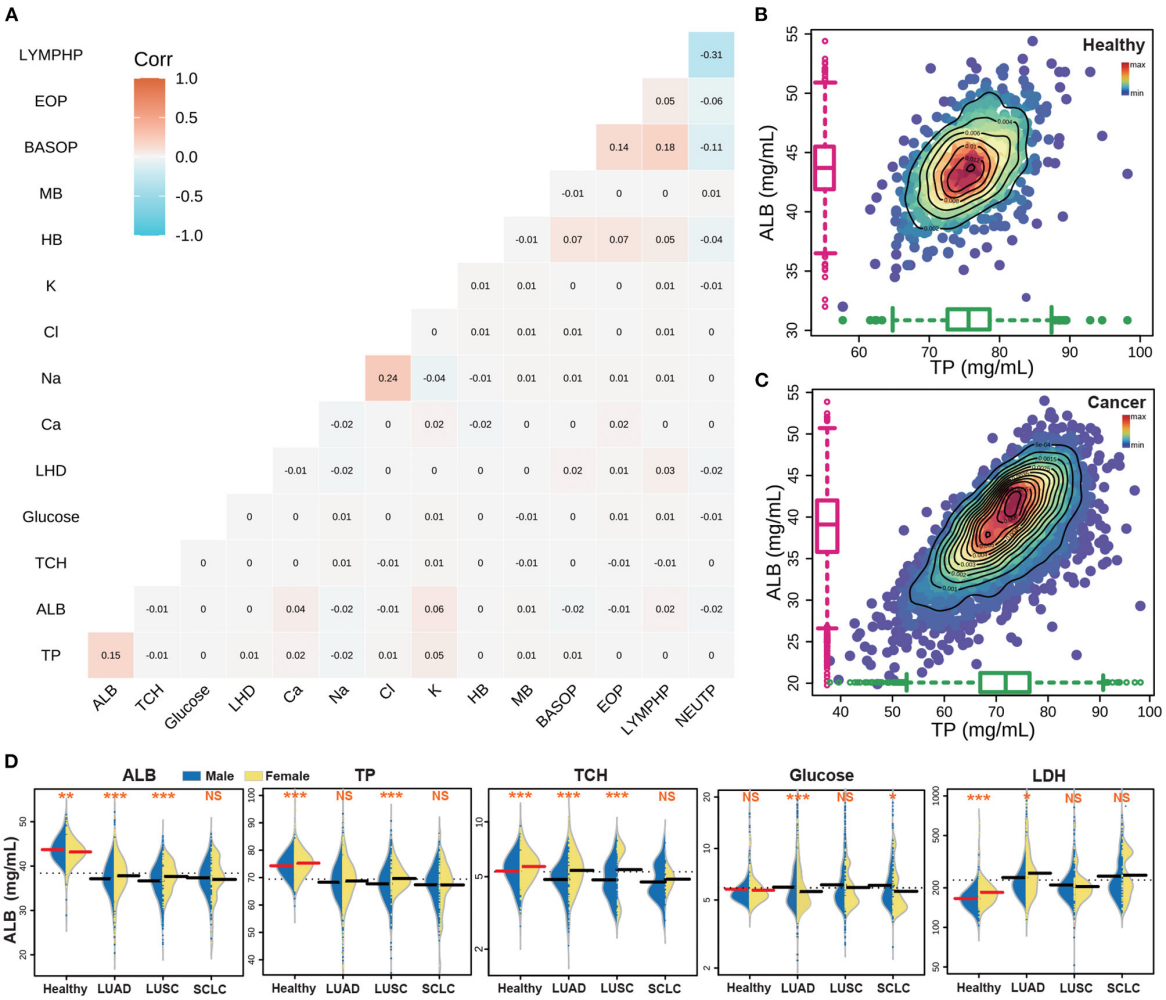
The results of the key nutritional indicators of cancer patients. **(A)** The correlation analyses with 15 nutrition indicators. **(B)** The correlation between ALB and TP in the healthy population. **(C)** The correlation between ALB and TP in lung cancer patients. **(D)** The distribution of five key nutrition indicators in different populations.

Based on previous studies of nutrition and ROC predict index, we selected 15 indexes as the objects of our research. Upon conducting correlation analysis of these indexes (Figure 3A), the highest correlations were found between Na and Cl, BASOP and LYMPHP, ALB and TP, and BASOP and EOP, which showed positive correlations. Conversely, there was a strong negative relationship between LYMPHP and NEUTP, and between BASOP and NEUTP. Furthermore, correlation analysis was performed on other indicators in the test data, and the results are shown in Supplementary Figure S2. The figure displays the distribution of correlation coefficients between lung cancer indicators, which may provide some reference value for the research of other projects. Of the five key nutritional indicators, the correlation coefficients of ALB and TP were the highest; thus, we plotted the scatter density diagram with TP as the abscissa and ALB as the ordinate (Figures 3B, C). The blue area indicates that the data points are scattered, while the red points represent the data set, delineated by contour lines of varying colors, which can directly visualize the density distribution among scattered points. Notably, healthy people were mainly concentrated in ALB: 42–46 mg/ml and TP: 73–78 mg/ml, while the index range of cancer people was concentrated in ALB: 36–42 mg/ml, TP: 68–76 mg/ml, indicating that both of these key nutritional indicators showed higher levels in healthy people than in those with cancer.

### 3.4. The statistical significance of nutritional indexes of subtype lung cancer patients

The great heterogeneity of lung cancer subtypes has been extensively documented (46). This heterogeneity is not only apparent at the genetic level, but it is also evident in the physiological indicators of the patient (47, 48). To this end, we have incorporated 15 patient-related indicators in our analysis to compare the biochemical index of healthy individuals and patients with different lung cancer subtypes. Moreover, we have observed that in general, the same indicators show differences between men and women patients (49), as exemplified in Figure 3D which depicts a comparison of the five key indicators between men and women. Additionally, we have performed a statistical analysis of the different indicators among different lung cancer subtypes, where a statistically significant difference is denoted by ***(*p* < 0.001), **(*p* < 0.01), and *(*p* < 0.05), and non-significance is denoted by NS. Our results showed that the content of ALB, TP and TCH in the healthy population was higher than that in the cancer subpopulation. Furthermore, the difference between males and females in the healthy population was highly significant. However, the difference between male and female indexes of large cell lung cancer was significant, while the difference in SCLC was the opposite, with no statistical significance between men and women. Additionally, the GLU content of healthy people was found to be similar to that of cancer patients, while the LDH content was lower than that of cancer patients.

**Figure 4:**
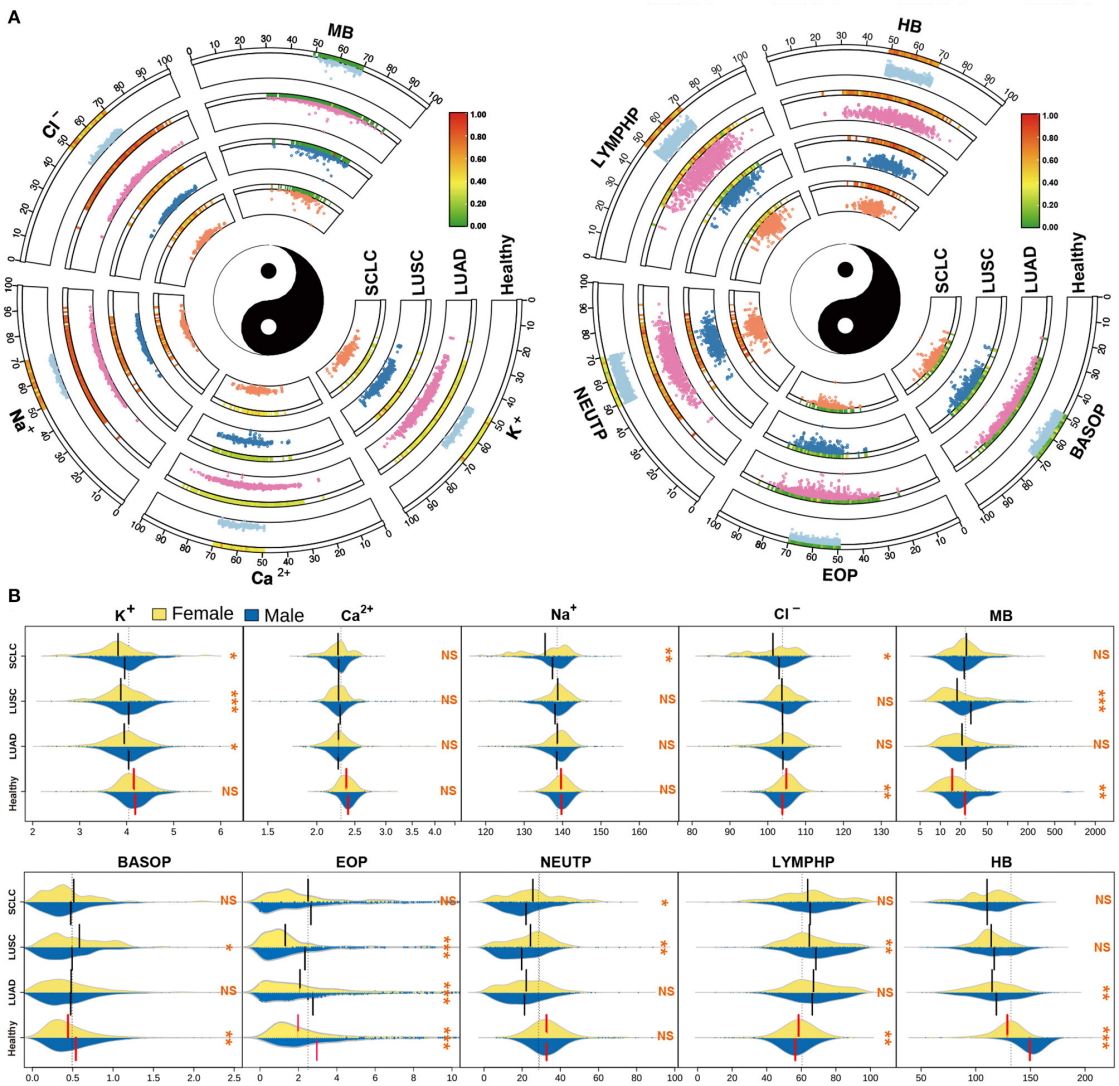
Basic information and index analysis of lung cancer patients. **(A)** The scatter distribution of age vs electrolytes and granulocytes indicators for the subtype of lung cancer patients. **(B)** The distribution of electrolytes and granulocytes indicators in different populations.

For further insight into the differences between the subtypes of lung cancer and the healthy population, we visualized the scatter distribution of age and index content for LUAD, LUSC and SCLC subtypes of lung cancer, and compared the distribution of healthy people (Figure 4A). The Circos diagram shows the age of patients between 0 and 100, and the vertical space in the sector box represents the relative content value of the indicators. The heat map bar displays the density of the scattered distribution of the index content of patients at different ages. The results demonstrate that there are some differences in the distribution of indicators in different subtypes of cancer. However, it is impossible to accurately quantify the indicators through the distribution of scattered points. To further analyze the differences between the subtypes of lung cancer and the healthy population, we also visualized the bean plot with the electrolyte and white blood cell index group (Figure 4B). The results indicate that there are significant differences between male and female indicators in healthy people. Additionally, we observed that there are no significant differences in the content of electrolytes between male and female medical records of subtypes of lung cancer, while most of the white blood cell indicators are statistically significant. Furthermore, healthy people generally have very significant differences which are consistent with the key nutritional indicators.

In this study, we conducted an overall analysis of lung cancer subtypes and compared their index content distribution. We observed significant differences between healthy individuals and different subtypes of lung cancer. Notably, there was no significant difference in key nutrition indexes of ALB, TP, TCH, and GLU between NSCLC and SCLC (Supplementary Figure S3). However, electrolyte indexes between the subtypes of lung cancer showed comparatively large differences. Furthermore, except for MB, BASOP and EOP, most other indicators showed highly significant differences between healthy individuals and cancer patients. Finally, LUSC and SCLC also exhibited significant differences in a majority of indicators. The analysis of these differences enables us to effectively differentiate between lung cancer subtypes and healthy groups.

### 3.5. Interrelation between nutritional indicators

**Figure 5:**
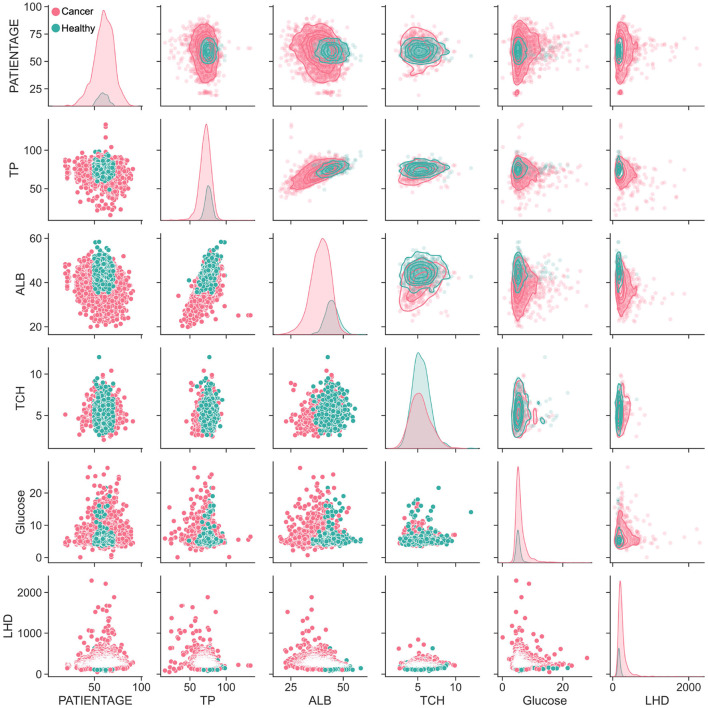
The correlation analysis between lung cancer patients and healthy population. Pairwise parameter combination analysis of the main nutritional indicators was conducted to visualize the distribution differences between cancer patients and ordinary patients.

The human body's digestion, absorption and metabolism are intricately connected (50). In order to properly analyze the nutritional indicators present in the body, a correlation analysis was conducted. Additionally, the distribution of 15 nutritional indicators was compared between healthy individuals (green points) and cancer patients (red points). Through this comparison, it was observed that the content level of the five key nutritional indicators for healthy individuals were mostly contained within the area of cancer patients (Figure 5). The lower section of the result depicted the scattered distribution of the indicators in the subject population, while the upper part represented the concentrated distribution. The diagonal line, on the other hand, depicted the density distribution of a single indicator. It was further observed that the healthy individuals' TP, ALB and TCH were located in the upper half of the distribution of lung disease population.

Moreover, the distribution relationship between different indicators was also analyzed. TP and ALB had a positive, linear correlation. Interestingly, when the TCH index was combined with other indicators, it was found that the lung cancer population was mostly covered by healthy people. Furthermore, all 15 indicators were analyzed and the trends between the distribution of five key indicators and other indicators were found to be generally consistent (Supplementary Figure S4). While the five key indicators mainly showed an elliptical distribution, the electrolyte index was more similar to a triangular distribution. This was because the proportion of granulocytes was used. Thus, the majority of the indicators showed an inverse linear distribution with granulocytes, meaning that the higher the proportion of granulocytes, the lower the related indicators.

### 3.6. Cancer prediction in random forest model

The ROC curve is a graphical technology that visualizes the performance of classifiers based on their performance (51). We used this technique to analyse the 15 selected nutrition-related indexes for a single indicator prediction model as shown in Supplementary Figure S1. We observed that some indexes such as GLU, BASOP and EOP had a poor predictive effect, with the AUC values far below 0.6. However, the AUC values of the other 12 indicators were all above 0.6, with ALB having the highest AUC value of 0.8. The overall ROC prediction performance for electrolytes was not very satisfactory, with Na ion and Cl ion having similar results. The indicators with a relatively good prediction effect were ALB, TP, TCH and HB.

**Figure 6:**
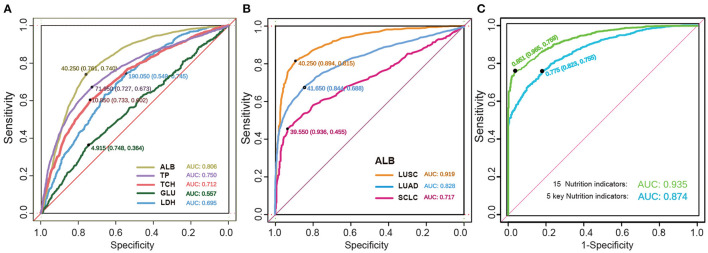
The detection of predictive effect of cancer models. **(A)** The ROC curve of the key biochemical index of nutrition in lung cancer. Indicators are shown by different colors, sensitivity, specificity, and AUC. **(B)** The ROC curve of the ALB in subtype lung cancer. **(C)** The ROC curve of the five key nutrition indicators and the whole 15 nutrition indicators.

Next, we used the tSNE visualization to compare the overall indicators. We used a machine learning algorithm to analyse the healthy population and lung cancer patients, and then obtained the ROC curve and calculated the AUC values of each indicator. As shown in Figure 6A, we grouped the ROC curve results of five important single index models. It was observed that the ALB had the highest accuracy among the five indicators, with an average index of 40.25 mg/ml, a sensitivity of 0.761, a specificity of 0.740, and an AUC of 0.806. The other indicators had a slightly lower performance, but still acceptable. However, the AUC of GLU was 0.557 and had almost no predictive ability.

We further investigated the ROC predictive curve of ALB in different subtypes of lung cancer, as shown in Figure 6B. In the LUSC subtype, the model had a very good predictive ability, with an AUC of 0.919. The sensitivity increased from 0.74 to 0.815, and the specificity increased to 0.894. Among the two NSCLC subtypes, LUAD had a lower predictive performance, with an AUC of 0.828 and a specificity of 0.844. However, the sensitivity decreased significantly. In SCLC, the AUC decreased to 0.717, and the sensitivity decreased to 0.455. Therefore, the ROC curve can effectively distinguish the difference in indicators between SCLC and NSCLC.

Finally, the AUC of the RF model was verified by combining the five key nutritional indicators with the 15 overall nutritional indicators, as shown in Figure 6C. The model showed a satisfactory performance in both the training cohort and verification cohort, with a sensitivity of 75.5% and 75.9%, respectively, and a specificity of 82.3% and 96.5%, respectively. These findings indicate that the forest-based random prediction can provide an alternative biopsy method with high specificity for lung cancer patients.

## 4. Discussion

In the present study, we identified nutrition indicator differences related to lung cancer prognosis by data statistic analysis. We collected 6,336 lung cancer patient data spanning a period of 5 years for statistical analysis, to determine the nutritional differences related to the prognosis of lung cancer and compare the differences between the relationship between different indicators and the subtype of lung cancer. Subsequently, we applied a Random Forest (RF) model for nutrition-based prediction of lung cancer prognosis, which proved to be feasible and had a high accuracy.

Our research population was dominated by patients aged 50–70 and above, accounting for 75.75% of lung cancer patients, which is consistent with the observation that the incidence of lung cancer increases with age (52, 53). In addition, the number of SCLC is significantly lower than NSCLC (46), which is also consistent with the fact that NSCLC accounts for 85% of cancer statistics (25). As clinical stage at the diagnosis is the main prognostic factor for NSCLC therapies, the use of patient's biochemical indicators and nutritional indicators to help predict whether the patient is diagnosed with cancer is essential in early detection (54). Therefore, we used the patient's biochemical indicators and nutritional indicators for predicting the prognosis of lung cancer. We defined the ALB index as the prognostic nutritional index (PNI) and inflammatory index of advanced lung cancer (55), which are key determinants of the prognosis of patients with solid tumors. Similarly, many studies also use the ratio of albumin to globulin (GLB) as a predictor (56–58), and the TP value is the sum of ALB and GLB. We show the density distribution relationship of the total protein of albumin in the results (Figures 3B, C). The distribution of cancer patients tends to be linear, and the cancer indicator is lower than that of healthy people. From the results of index analysis (Figure 3D), the results indicated that the ALB, TP and TCH of healthy people are significantly higher than those of cancer patients. Cancer patients are deprived of more nutrients by cancer cells to supplement the growth of cancer, thus relatively healthy people consume more energy and nutrients to maintain their daily needs.

Cancer patients have a more complex microenvironment, which affects the hormone balance of patients (59). Consequently, the overall indicators of cancer patients basically cover the range of healthy people. In addition, the relationship between the two indicators also shows this trend. There are significant differences between lung cancer patients and healthy people, which can be well predicted by indicators. However, among lung cancer subtypes, the nutritional indicators do not show much difference. Therefore, it is also a big challenge to predict lung cancer subtypes through nutrition indicators.

Our predictive model was developed for the early stage and prognosis of lung cancer using a combination of nutritional indicators and a machine learning algorithm. We applied 70% of the data as training data, and the accuracy rates we achieved are all greater than 90%. Our model included 15 predictors, including ALB, TP, TCH, GLU, LDH, K^+^, Ca^2+^, Na^+^, Cl^−^, MB, BASOP, EOP, NEUTP, LYMPHP, HB. The prediction accuracy of the single predictor is shown in Supplementary Figure S1. Furthermore, the combination of the indicators can get better accuracy. Consequently, the AUC for the 15-index-RF model was as high as 93.5%. Five key nutrition indicators (ALB, TP, TCH, GLU, LDH) have been regarded as the predictor correlated with lung cancer (5–9). In our study, the accuracy of the 5-index-RF model was as high as 87.4%. Granulocyte and neutrophil lymphocyte ratio, Na, Cl, K, and Ca ion homeostasis are also associated with tumor development and metastasis (36, 38), and their serum concentrations are closely related to the overall survival of lung cancer patients, and should be considered as clinical prognostic factors. Therefore, our model showed that the combined indexes have high accuracy in predicting lung cancer.

## 5. Conclusion

There are significant differences between men and women in healthy individuals and common NSCLC, however there is no significant difference between men and women in SCLC patients. ALB and TP were considered as the most essential nutrition indicators, and the prediction result from a single indicator proposed that they had the most prominent impact on the prediction.

The accuracy obtained in the lung cancer predictions in our study was similar to or better than the results previously published. The average AUC with five key nutrition indicators was approximately 87.4% for all lung cancer predictions, while the AUC for the 15-index-model was as high as 93.5%, with a sensitivity of 75.9% and a specificity of 96.5%, which appears to be reasonable in many applications. This high specificity may make our method viable for screening and suggest that the prediction of the RF model can provide an adequate substitute for biopsy in lung cancer patients.

Furthermore, not like many other published results which focused on predicting particular diseases, the method of composite index prediction applied in our study can be used to predict the risk of any nutrition-related disease, since many diseases have the capacity to affect the patients' nutritional indicators. Ultimately, we perceive the results attained using the proposed prediction model to be suitable for our intended use, which is customized to health communications.

## Data availability statement

The original contributions presented in the study are included in the article/Supplementary material, further inquiries can be directed to the corresponding authors.

## Author contributions

HL lead this research, conducting the data analysis, and writing the manuscript. ZC was responsible for the visualization of figures and the revision of the manuscript. BS provided supervision, validation, and funding support. JL and CX offered guidance on research methods and proofreading cancer research theories. ZL organized the data and visualized part of the data. ML and LL collected and screened cancer patient data and healthy population samples. ZS typeset the resulting drawings and manuscript. All authors contributed to the manuscript and gave their approval for its submission.

## Supplementary material

The contents in the supplementary figure are all supplements to the viewpoints and information in the paper, some of the multi-indicator maps cannot be directly put into the main figure of the text, so they are presented in the supplementary material, and the expanded contents of this study are discussed more comprehensively.

In the Supplementary material, Supplementary Figure S1 comprehensively shows the ROC curves and corresponding AUC values of 15 nutrition-related indicators. Supplementary Figure S2 shows the correlation analysis results of more than 40 biochemical indicators. Supplementary Figure S3 shows the content distribution of 15 nutritional indicators of the whole lung cancer subtype and the analysis results of the different significance between the subtypes. Supplementary Figure S4 shows the scatter distribution of 15 nutritional indicators.

Supplementary Figure S1 The ROC curve of single indicator prediction model. The predictive effect of 15 single index prediction models can be quantified by AUC to quantify the efficiency of model prediction.

Supplementary Figure S2 The heatmap of correlation analysis of blood biochemical indexes. The heat map of the correlation analysis between 50 biochemical indicators. The correlation value is −1 to 1, with negative values being negative and positive values being positive, and the higher the value, the greater the correlation.

Supplementary Figure S3 The statistical significance analysis of subtype lung cancer. The statistical significance analysis of indicators with electrolytes, nutritional indicators and granulocyte ratios.

Supplementary Figure S4 The paired relationship between biochemical index with lung cancer patients and healthy population. The correlation analysis of pairwise parameter combination analysis of the 15 nutritional indicators was conducted to visualize the distribution differences between cancer patients and ordinary patients.
