# Supplementary figures and images for: Novel nutritional indicator as predictors among subtypes of lung cancer in diagnosis
Source: Front Nutr. 2023 Jan 26;10:1042047. doi: 10.3389/fnut.2023.1042047 (PMC9909296; doi:10.3389/fnut.2023.1042047)

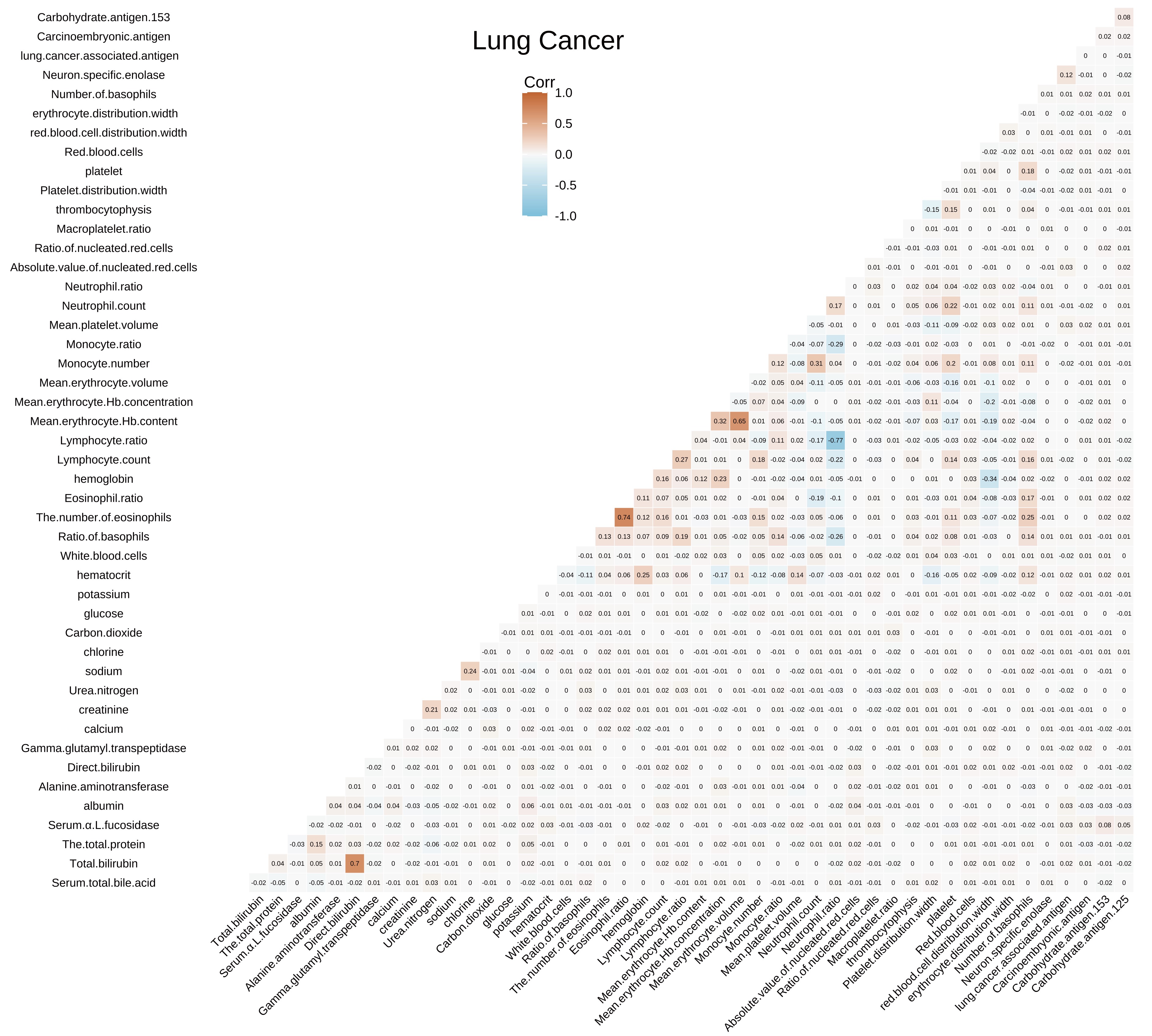

Supplement: Supplementary Figure S2 — The heatmap of correlation analysis of blood biochemical indexes. The heat map of the correlation analysis between 50 biochemical indicators. The correlation value is −1 to 1, with negative values being negative and positive values being positive, and the higher the value, the greater the correlation. [file Image_2.JPEG]
